# Off pump coronary artery bypass surgery in a Nigerian teaching hospital

**DOI:** 10.11604/pamj.2013.14.122.2255

**Published:** 2013-03-28

**Authors:** Michael Sanusi, Bode Falase, Salisu Ismail, Adetinuwe Majekodunmi, Adeyemi Johnson, Ifeoluwa Ajose, David Oke

**Affiliations:** 1 Cardiothoracic Division, Department of Surgery, Lagos State University College of Medicine, Lagos State University Teaching Hospital, Lagos, Nigeria; 2 Department of Anaesthesia, Lagos State University College of Medicine, Lagos State University Teaching Hospital, Lagos, Nigeria; 3 First Cardiology Consultants, Lagos, Nigeria; 4 Cardiology Division, Department of Medicine, Lagos State University College of Medicine, Lagos State University Teaching Hospital, Lagos, Nigeria

**Keywords:** Coronary artery bypass surgery, off pump, left internal mammary artery, left anterior descending coronary artery, Lagos, Nigeria

## Abstract

Coronary Artery Bypass Grafting has not been previously reported in the Nigeria Medical Literature. We report the case performed in our institution of a 56 year old Nigerian female who underwent Off Pump Coronary Artery Bypass Surgery (OPCAB) for an ostial lesion of the Left Anterior Descending Coronary Artery. The Left Internal Mammary Artery was successfully anastomosed to the Left Anterior Descending Coronary Artery. The patient was discharged home after 2 weeks, following correction of problems with glycemic control.

## Introduction

Off Pump Coronary Artery Bypass Surgery (OPCAB) is myocardial revascularization on a beating heart without the aid of the Cardiopulmonary Bypass Machine. This procedure is now popular in many cardiac centres worldwide [1]. There is however a paucity of reports of this procedure in West Africa, with no report from Nigeria.

## Patient and observation

**Figure 1:**
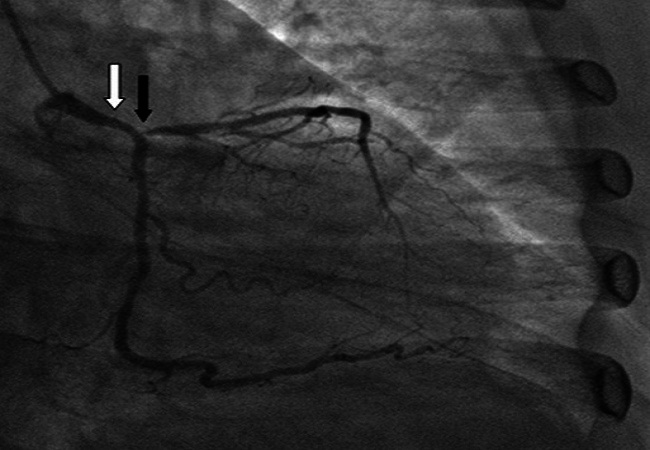
Coronary Angiogram showing Left Main Stem stenosis (white arrow) and proximal Left Anterior Descending Coronary Artery stenosis (black arrow)

The patient referred to our unit for consideration for coronary artery bypass grafting was a 56 year old teacher who had been managed for Ischaemic Heart Disease (IHD) at a private cardiology facility in Lagos. She had presented in July 2009 with a history suggestive of IHD and angina class III (Canadian Cardiovascular society classification) which was worsening despite medical therapy. Coronary angiography done demonstrated significant lesions in the mid portion of the Left Descending Coronary Artery (LAD) and the proximal Circumflex Coronary Artery (Cx). The Right Coronary Artery was a dominant artery with some minor irregularities. Percutaneous transluminal coronary angioplasty of both the LAD and Cx was done. A 3.0 mm Drug Eluting Stent was deployed to stent the LAD and the Cx was stented with a 3.0mm Bare Metal Stent. The patient was angina-free for one year but represented in July 2010 again with angina class III. Repeat coronary angiogram was done which showed that both stents were patent and there was no new coronary lesion. She was controlled on medical therapy. However she presented again in November 2011, this time with unstable angina. An urgent coronary angiogram carried out showed that the previous stents were still patent but with a 50% left main stem stenosis and a 95% proximal LAD stenosis (Figure 1). She was subsequently referred for surgical revascularization.

Physical examination on admission revealed a middle aged woman who was clinically obese with a body mass index of 32kg/m^2^. There were no significant physical findings. Associated risk factors were intermittent claudication (Ankle-Brachial Index bilaterally was 0.57), bilateral carotid bruits, poorly controlled diabetes mellitus and hyperlipidemia. Her calculated euroscore was 6. Medications on admission were Aspirin, Glyceryl trinitrate sublingual spray, Metformin, Glibenclamide, Fluvastatin, Metoprolol and Isosorbide Dinitrate. Investigations done included transthoracic echocardiogram which showed good left ventricular ejection fraction with no evidence of ventricular dysfunction. Chest radiogram, 12 lead electrocardiogram and pulmonary function tests were normal. All blood parameters were within acceptable limits. Following review of her coronary angiogram she was scheduled for single vessel grafting of the LAD as an off pump procedure.

**Figure 2:**
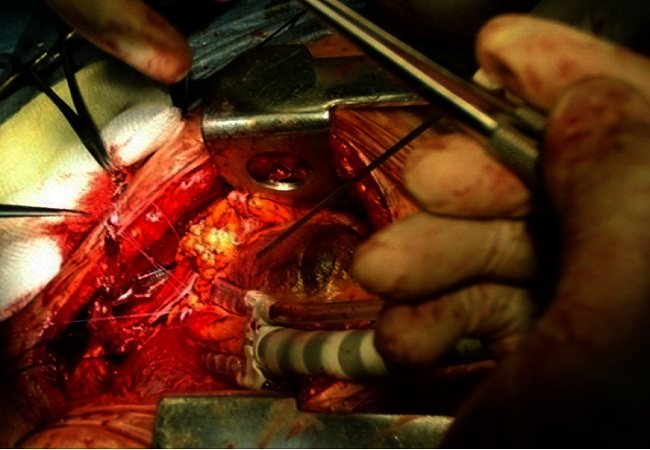
Left Internal Mammary Artery being anastomosed to the Left Anterior Descending Coronary Artery

Surgery was performed in November 2011. The cardiopulmonary bypass circuit was not primed. Following median sternotomy and harvesting of the Left Internal Mammary Artery (LIMA) the Octopus 3 Off-pump stabilizer and foot plate were used to immobilize the anterior myocardial surface with good visualization of the LAD. The LAD which was a 2.5mm vessel was snugged proximally and an arteriotomy performed in its mid-portion. The arteriotomy site was kept bloodless with CO2 insufflation via an improvised blow-mister. The LIMA to LAD anastomosis was performed with 6-0 prolene suture (Figure 2). The procedure was uneventful and the patient was transferred to the Intensive Care Unit on minimal inotropic support. She was weaned off the ventilator after 4 hours and inotropic support was discontinued after 24 hours. Postoperative recovery was delayed by the need to achieve glycaecmic control. She was discharged home 2 weeks postoperatively. She has been reviewed in clinic and remains free of angina.

## Discussion

Conventional on pump Coronary Artery Bypass Grafting (CABG) is done with the assistance of cardiopulmonary bypass (CPB) which is associated with an increased systemic inflammatory response which contributes to the morbidity from the procedure [2]. This morbidity is however reduced to a minimum with the introduction of OPCAB which is a valuable alternative means of surgical myocardial revascularization as by eliminating the inflammatory pump reaction it reduces the incidence of postoperative cardiac, pulmonary, renal and neurological dysfunction. It has been established that OPCAB leads to better clinical outcome than the on-pump approach in certain high risk subgroups like the elderly, diabetic patients, and those with poor preoperative vital organ reserve. The benefits include reduction in mortality and morbidity, reduction of length of hospital stay, as well as economic benefit [3].

Ischemic heart disease had initially been reported to be rare in the black African, representing only 6% of all cardiovascular diseases [4]. The reasons advanced for this include diet, lifestyle and genetic factors like more efficient homocysteine metabolism by the black African [5]. A recent study of epidemiological transitions shows that ischemic heart disease now ranks 8^th^ among the leading causes of death in the African region and is responsible for 14% of cardiac deaths. This is still well below the reported 50% of cardiac deaths being of ischemic origin in the western world [6]. With the increase in IHD, there is a growing need for appropriate facilities and expertise in performing Coronary Artery Bypass Surgery.

A review of the literature shows Open Heart Surgery being performed in 5 West African Centres (Ghana, Nigeria, Cote D'Ivoire, Senegal and Cameroon) [7]. There is no specific information in the sub-Saharan African literature about Off-Pump Coronary Artery Bypass Surgery (OPCAB) and there is limited information about CABG in general. In Ghana, CABG accounts for only 3.5% of the total number of open heart cases done [8]. There is only one publication in the English literature specifically addressing CABG in the West African sub-region [9].

OPCAB was initially introduced in the 1950s. Soon after, most Cardiothoracic Surgeons found the still and bloodless field afforded by CPB to be superior for performing coronary artery bypass grafting. Beating heart surgery was for the most part abandoned. With the improvement of technology and use of mechanical stabilization techniques, the popularity of the Off-Pump procedure has increased. Multiple reports from institutions performing large numbers of OPCAB have confirmed the safety and efficacy of this procedure.

There is a significant learning curve with OPCAB which stems from familiarity with conventional CABG. Single vessel grafting as performed in our patient is therefore advisable as an initial starting point for a program just developing the expertise.

In the Western World, OPCAB costs less than conventional CABG for low risk patients, with a cost savings of 1,375 US dollars [10]. As Open Heart Surgery facilities develop in Nigeria and more procedures become available locally, the affordability of the cost of coronary artery bypass grafting will be one of the factors that determine if OPCAB or conventional CABG takes root in Nigeria. The actual cost of this procedure in our institution was 8,440 US dollars, which compares favorably with the cost in other cardiac centres.

## Conclusion

In sub-Saharan Africa there is an increasing incidence of IHD which requires the development of facilities and expertise for Coronary Artery Bypass Surgery. The advantages of OPCAB over conventional CABG may make it a better approach to myocardial revascularization in our environment, especially in higher-risk patients like our reported patient who have multiple co-morbidities.
